# Time to development of surgical site infection and its predictors among general surgery patients admitted at specialized hospitals in Amhara region, northwest Ethiopia: a prospective follow-up study

**DOI:** 10.1186/s12879-023-08301-0

**Published:** 2023-05-17

**Authors:** Meron Asmamaw Alemayehu, Abebaw Gedef Azene, Kebadnew Mulatu Mihretie

**Affiliations:** 1 Department of Epidemiology and Biostatistics, Institute of Public Health, College of Medicine and Health Sciences, University of Gondar, Gondar, Ethiopia; 2 Department of Epidemiology and Biostatistics, School of Public Health, College of Medicine and Health Sciences, Bahir Dar University, Bahir Dar, Ethiopia

**Keywords:** Surgical site infection, General Surgery, Time to development, Survival time, Amhara region, Ethiopia

## Abstract

**Background:**

Surgical site infection is an infection occurring within 30 days after surgery. It is recently reported that evidence-based information on the specific time when the majority of surgical site infections would develop is a key to early detect the infection as well as to preventing and early intervene against their pressing and fatal complications. Therefore, the current study aimed to determine the incidence, predictors, and time to development of surgical site infection among general surgery patients at specialized hospitals in the Amhara region.

**Method:**

An institution-based prospective follow-up study was conducted. The two-stage cluster sampling procedure was used. A systematic sampling technique with a K interval of 2 was applied to prospectively recruit 454 surgical patients. Patients were followed up for 30 days. Data were collected using Epicollect5 v 3.0.5 software. Post-discharge follow-up and diagnosis were done by telephone call follow-up. Data were analyzed using STATA™ version 14.0. Kaplan–Meier curve was used to estimate survival time. Cox proportional regression model was used to determine significant predictors. Variables with a *P-value* less than 0.05 in the multiple Cox regression models were independent predictors.

**Result:**

The incidence density was 17.59 per 1000 person-day-observation. The incidence of post-discharge Surgical site infection was 70.3%. The majority of surgical site infections were discovered after discharge between postoperative days 9 to 16. Being male (AHR: 1.98, 95% CI: 1.201 – 3.277, diabetes Mellitus (AHR: 1.819, 95% CI: 1.097 – 3.016), surgical history (AHR: 2.078, 95% CI: 1.345, 3.211), early antimicrobial prophylaxis (AHR: 2.60, 95% CI: 1.676, 4.039), American Society of Anesthesiologists score ≥ III AHR: 6.710, 95% CI: 4.108, 10.960), duration of the surgery (AHR: 1.035 95% CI: 1.001, 1.070), Age (AHR: 1.022 95% CI: 1.000, 1.043), and the number of professionals in the Operation Room (AHR: 1.085 95% CI: 1.037, 1.134) were found to be the predictors of time to development of Surgical site infection.

**Conclusion:**

The incidence of surgical site infection was higher than the acceptable international range. The majority of infections were detected after hospital discharge between 9 to 16 postoperative days. The main predictors of Surgical site infection were Age, Sex, Diabetes Mellitus, previous surgical history, the timing of Antimicrobial prophylaxis, American Society of Anesthesiologists score, pre-operative hospital stay, duration of surgery, and the number of professionals in the operation room. Hence, hospitals should give great emphasis on pre-operative preparation, post-discharge surveillance, modifiable predictors, and high-risk patients, as they found in this study.

## Introduction

Surgical site infection (SSI) is one of the most common types of hospital-acquired infections in developing countries. It is defined as an infection occurring within 30 days after an operative procedure is performed. The types of SSI are defined y using a set of standard clinical diagnostic criteria and the CDC America diagnostic criteria is the most commonly used one. According to the CDC America, SSI can be superficial, deep soft tissue or organ/space [1].

There are several ways SSI can be caused. The majority are caused by an endogenous infection, which is when the incision becomes contaminated with microorganisms derived from the patient’s skin or an opened internal organ. Exogenous infection occurs when external microorganisms contaminate the operative site during the procedure. Sources include surgical instruments, the theatre environment, and the air. External microorganisms can also contaminate the wound at the time of an incident, or gain access to the wound following surgery before the wound has healed. Causative pathogens depend on the surgical site; for example, the risk of developing SSI from enteric gram-negative microorganisms increases with surgery on the gastrointestinal tract [2].

SSI is the 3rd commonly reported nosocomial infection accounting for 10 to 40% of all nosocomial infections [3]. Globally, SSI rates range from 2.5% in Europe [4] to 41.9% in Africa (Tanzania) [5]. There are local and systemic complications of SSI. Spontaneous wound breakdown/dehiscence is the commonest local complication while bacteremia with the possibility of spread and sepsis are the most important systemic complications. These complications are entirely preventable if there is a way to early detect the SSI and initiate treatment as early as possible [6]. However, a recent study indicated that about 20–60% of surgical patients develop those preventable complications because the majority of cases occur after patients are discharged from the hospital. If not treated urgently, SSI complications often require secondary surgery and are related to a 3.2% of fatality rate [6, 7].

According to the International Surgical Wound Complications Advisory Panel (ISWCAP) 2020 report, even if SSIs are expected to occur within 30 days after surgery, the specific time when the majority of them would develop is key to early detect SSI and thus, to prevent and early intervene against their pressing and fatal complications. Knowing the specific time is also very important to reduce the likelihood of wound complications, such as bacteremia, progressing to a more complex situation [7].

In Ethiopia, there is ample research on the overall rates, causes, and costs of SSI [8, 9]. However, no attention has been paid to the time when the majority of SSIs occur and to the rate of post-discharge SSIs. Existed studies in the country reviewed only hospital records and followed patients merely up to discharge to find SSI cases, which vastly underestimated the true size of the problem. This indicates that the paramount problem of previous studies in the country is not only related to the neglection of post-discharge SSI cases but also the lack of time. Therefore, robust methods of identifying infections after patients have been discharged from the hospital are critical and will find more cases of infection than retrospective or passive methods [4, 10].

Recent studies in other countries revealed that 70% of SSIs are detected post-discharge and the median hospital length of stay (LOS) for surgical patients is becoming shorter and shorter than ever. Consequently, hospitals in those countries are initiating different programs to trace post-discharge SSI cases, to reduce the infection rate as well as to prevent its complications [4, 10, 11]. One of these programs being implemented is post-discharge SSI surveillance and control activity. Hospitals with this effective program have reported a reduction of SSI rate by 32% and complications by 48% [11]. A Few programs implemented but found to be ineffective for hospitals in developing countries include; preparing an infection control physician, one infection control nurse per 250 beds, and a system for reporting infection rates to practicing surgeons. It has been reported the reason for this was those programs are highly resource intensive to be constantly implemented in developing countries [12].

Effective infection control and prevention programs like post-operative surveillance do not exist at specialized hospitals in the Amhara region. Studies like this can increase awareness and insight into the importance of and the need for those program activities. Therefore, this study aimed to estimate the survival time and predictors of surgical site infection among surgical patients at specialized hospitals in the Amhara region.

## Methods and materials

### Study area, design, and period

An institution-based prospective follow-up study was conducted between March 2021 to April 2021, to assess the time to development of surgical site infection and its predictors among general surgery patients at specialized hospitals in the Amhara region. The region is the 2^nd^ largest and most populous in Ethiopia. It has eight specialized hospitals and the study was conducted in three (randomly selected) specialized hospitals namely: Felege Hiwot Comprehensive Specialized Hospital (FHCSH), University of Gondar Comprehensive Specialized Hospital (UoGCSH), and Tibebe Ghion Specialized Teaching Hospital (TGSTH). FHCSH and TGSTH are located in Bahir Dar City, the capital city of the Amhara National Regional State. The Bahir Dar city is 565 km far from Addis Ababa (the capital city of Ethiopia). FHCSH has 12 wards with 42 departments. The hospital has a total of 477 functional beds. Of these functional beds, 100 belong to the surgery ward and the median length of stay in the ward is 6.5 days. Likewise, TGSTH has 11 departments and 461 functional beds, of these, 104 beds are found in the surgical ward. The median LOS of the surgical ward in this hospital is 6 days. [13–15]. UoGCSH is the oldest hospital in the country and is located in Gondar city, 185 km north of Bahir Dar city. It has 11 departments with 400 functional beds and 96 beds belong to the surgery ward. The median length of stay in the surgery ward is 6 days.

### Sampling techniques and procedure

**Fig. 1:**
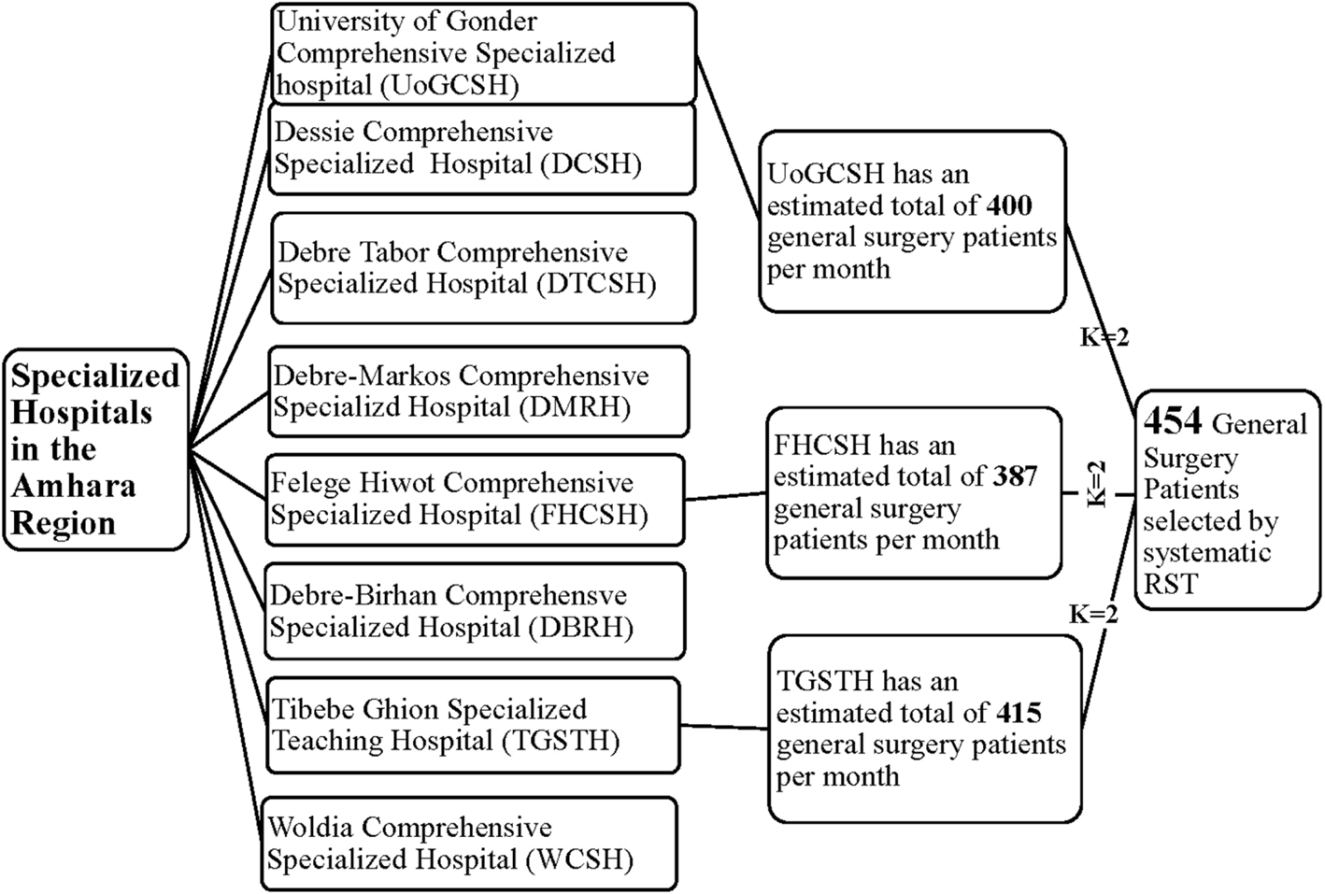
Sampling procedure for survival time and predictors of SSI among general surgery patients in Amhara Region, 2021 G.C

The sample was obtained using a two-stage cluster sampling technique. The first stage involved selecting a random sample of 3 hospitals from the 8 specialized hospitals in the region. In the second stage, a random sample of post-operative patients was obtained from each hospital by using a systematic random sampling technique with a sampling interval of (k = 2) (Fig. 1). K was determined by dividing the total estimated general surgery patients per month in the three hospitals (400 + 387 + 415), by the calculated sample size (*n* = 454). The total number of general surgery patients per month in each hospital was estimated by taking the average of the total number of general surgical patients flow for the past 12 months. For those patients who agreed to participate and fulfill the inclusion criteria, a unique study ID number was given immediately after their surgery. The population in this study was assumed to be homogeneous and the surgical capacity (per day) of the three study hospitals was relatively equal. As a result, there was no need to proportionally allocate the sample size for the three study hospitals. Instead, general surgery patients at a K interval of 2 were enrolled in the study until the calculated sample size (454) was fill-up. Since this was an open-prospective study (i.e., new surgical patients recruited into the study at any time during the follow-up), there was no sampling frame.

### Population

The source populations are all surgical patients who underwent general surgery from 2020 until the end of 2021. The study population was patients admitted to either of the three study hospitals namely, UoGCSH, FHCSH, and TGSTH who have undergone general surgical procedures from March to April 2021 G.C. Study units were patients who underwent a general surgical procedure in the study hospitals from March to April 2021 G.C.

### Sample size determinations

The sample size was calculated using Stata™ Version 14.0 (sample size analysis for Cox proportional hazards model) by considering hazard ratio of SSI 1.48 (pre-operative hospital stay > 72 h), two-tailed significant level (α) of 0.05, power 80%, and a 95% confidence level (Table 1).

**Table 1:** Sample size determination for time to development of surgical site infection and its predictors among general surgery patients at specialized hospitals

Variable	Assumptions			Sample size	Reference
	HR	Event	P (e)		
Duration of the surgery > 120 min	2.57	36	0.2	188	[16]
Pre-operative hospital stays > 72 h	1.48	204	0.5	454	[17]

### Eligibility criteria

All patients with age ≥ 1 year, admitted for elective or emergency, clean and clean-contaminated surgery were included in the study. Patients who underwent implant surgeries, patients whose wounds were not primarily closed in the operation room, and patients who underwent re-surgery of the same site during the follow-up were excluded from the study.

### Study Variables

The twenty-four independent variables included in the study were; Age, Sex, Occupational status, Educational Status, Residence, BMI, DM, HIV/AIDS, Other Comorbidities, Alcohol Drinking, Cigarette Smoking, History of Previous Surgery, Type of surgical procedure, Grade of the Surgeon, Preoperative Blood Transfusion, American Society of Anesthesiologists (ASA) score, Length of Pre-operative hospital stays, Wound contamination class (clean, clean-contaminated), Duration of the operation, The urgency of the surgery (elective or emergency surgery), Timing of antimicrobial prophylaxis, Presence of Drain, Number of professionals in the OR, and Presence of wound care.

### Operational /Term/ Definitions

Event: was the development of the first surgical site infection following a particular general surgery procedure.

Start time: was post-operative day one (i.e., the procedure day), while, the end time was Post-operative day 30.

Time to Surgical site infection: time (in days), between the end of the operation to the development of surgical site infection following general surgery.

#### Censoring 

Patients who died during the follow-up without developing SSI, patients referred to other hospitals, patients who do not develop SSI until postoperative day 30, Non-response patients (after five calls), patients whose phone did not work after 3 consecutive days of trial (lost to follow-up), and patients who refused to participate during the follow-up.

#### Superficial SSI

Patients were considered as having superficial surgical site infection if; the date of the event for infection occurs within 30 days after the surgical procedure (where day 1 is the procedure date) and involves only skin and subcutaneous tissue of the incision and the patient has at least one of the following:

A Purulent drainage from the superficial incision.
B A superficial incision that is deliberately opened by a surgeon or attending physician to manage the infection and the Patient has at least one of the following signs or symptoms of infection: fever (> 38 °C); pain or tenderness; localized swelling; offensive or non-offensive purulent discharge, erythema or heat [18].

#### Deep incisional SSI

Patients were considered as having deep incisional SSI if; the date of the event for infection occurs within 30 days after the surgical procedure (where day 1 is the procedure date) and involves deep soft tissues of the incision (for example, deep abscess of muscle or facial layer) and the patient has at least one of the following:

A Purulent drainage from the deep incision.
B Spontaneous dehiscence or deep incision that is deliberately opened by a surgeon or attending physician to manage infection.
C At least one of the following symptoms: fever (> 38 °C); localized pain or tenderness [19].

#### Diagnosis of Organ/Space SSI

Patients were considered as having organ/space SSI if; the date of the event for infection occurs within 30 days after the surgical procedure (where day 1 is the procedure date) according to the list that can be found at [20] Infection involves any part of the body deeper than the fascial/muscle layers, that is opened or manipulated during the operative procedure and the patient has at least one of the following:

A Purulent drainage from the drain that is placed into the organ/space (for example, closed suction drainage system, open drain)
B An organism is identified from an available microbiologic testing method which is performed for purpose of clinical diagnosis or treatment [20].

General Surgery:—is a surgery that focuses on abdominal and thoracic contents including the esophagus, stomach, small intestine, large intestine, liver, pancreas, gallbladder, appendix and bile ducts, and often lung and thyroid gland. It also does include fistula surgeries such as anorectal, vaginal, or other fistula surgeries [21].

Current Smokers: are patients who were smoking a cigarette at the time of the study [22].

Former Smokers: are patients who have smoked cigarettes but quit at the time of the study [22].

Never Smokers: are patients who have never smoked cigarettes [22].

### Data collection tool and procedure

A validated data collection tool was adapted from ‘WHO Protocol for Surgical Site Infection Surveillance with a Focus on Settings with Limited Resources’ and it was amended according to local circumstances. Data were collected using Epicollect5 version 3.0.5 by 2 trained BSc Nurses (for each hospital) not working in a surgical ward. At discharge, all patients were asked to provide one or more telephone numbers, either a personal phone and/or that of their attendant for communication after discharge. Health information was given to every patient/attendant and clear instruction and training was delivered to help them actively report any signs and symptoms of surgical site infection. They were also emphasized to check the incision site at least twice daily for any signs and symptoms of infection.

There were two major time periods in this study. Perioperative (in-patient) time and out-patient (post-discharge) time. During the in-patient data collection, each participant had one form per operation. In order to gain the most accurate data and allow encourage surgical staff to record all the appropriate information in the patient notes, the peri-operative data collection form was completed before and during the surgery, when it is possible. When surgery takes place overnight, when the data collectors are not in the hospital, following this up was the first task in the morning. Information was obtained directly from patients, operation notes, medical and medication charts as well as direct observation (when possible). The data collectors followed the patients and reviewed their charts every three days after the operation until the patients were discharged from the hospital, fail, or Censored. Wound classification was done using the Center for Disease Prevention and Control (CDC) criteria for surgical site infection surveillance.

For those patients discharged before the 30^th^ post-operative day without developing surgical site infection, the second period of data collection (post-discharge data collection) began at their discharge and it stayed until the patient develop SSI or was censored. All participants were told they will receive follow-up calls every 3 days about their surgical wound and thus, to check the incision site at least twice daily for any signs and symptoms of infection. Participants were communicated using the mobile or home phone number they had provided at discharge.

During each phone call communication, a brief, standard, and structured series of interviews regarding the current status of the wound were conducted by using a validated ‘WHO surgical site infection post-discharge data collection form’. Data collector nurses asked questions to distinguish between the presence of expected postoperative wound changes or signs/symptoms suggestive of SSI. The most common SSI suggestive sign and symptoms are the following. Visible green/yellow pus or discharge with or without bad smell coming from the wound (a small amount of reddish-brown drainage is common for 1 or 2 PODs); heat of the incision area or surrounding skin (hot to touch wound); unexplained or increasing pain or tenderness at the surgical wound that is beyond normal for operation (pain that does not relieve by bed rest or by NSAIDs such as Diclofenac, ibuprofen, and paracetamol and/or patient report > 6 on the self-pain rating scale); the presence of spontaneous gaping/breakdown of the wound with or without protrusion of internal structures; localized swelling; or redness of the wound (*Annex*). A series of ‘yes’ or ‘no’ questions were also asked to diagnose SSI in patients who noticed any changes in their wounds. This post-discharge telephone call communication was designed to represent a form of clinical interaction and had an appropriate degree of politeness, sensitivity, and confidentiality.

When a patient or attendant reported the presence of unambiguous signs and symptoms (i.e., previously mentioned), SSI was diagnosed with only a phone call examination. However, to diagnose SSI in situations where the patient/attendant was not sure about the absence of SSI suggestive signs and symptoms, the diagnosis was done in three different strategies. *First*, patients were asked to go to the nearby health center and check their wound status. *Second*, patients were asked if their outpatient department (OPD) appointment for their surgical wound was closer to that day, and if it was closer, the diagnoses had been made after their (OPD) appointment examination. The OPD appointment of discharged surgical patients in the study hospitals was usually 2 weeks after discharge. *Third*, a liaison with a health extension worker was created, where available, and the data collectors inquired the HEWs about the patient's condition and incision site. The day when patients reported developing the first sign or symptom of SSI was recorded as their failure time.

The accrual period was estimated by dividing the total required sample size (*n* = 454) by the sum of the expected rate of general surgery (ERGS) per day from each hospital ($$\frac{n}{ERGS per day at hospital 1 + ERGS per day at hosptial 2 +ERGS per day at hospital 3}$$). The expected rate of general surgery (ERGS) per day was estimated by considering the information from the hospitals. According to information from FHCSH, on average, 13 general surgeries per day (9 elective and 4 emergency surgeries) would be done. In TGSTH, on average, 16 general surgeries per day (11 elective and 5 emergency surgeries) would be done. In UoGCSH, on average, 15 general surgeries per day (12 elective and 3 emergency surgeries) would be done. Therefore, by expecting about 10 eligible patients per day from FHCSH, 13 eligible patients per day from TGCSH, and 13 eligible patients per day from UoGCSH, the accrual period was estimated to take about 13 days ($$\frac{454}{10+13+13 })$$. However, it took a total of 22 days to prospectively recruit the 454 samples.

Data were collected by two trained BSc Nurses (in each hospital) not working in the surgical ward. The data collection tool was pretested after being carefully adapted according to local/hospital circumstances. The pre-taste was done with patients equivalent to 5% [23] of the calculated sample size at Addis Alem Hospital, a primary Hospital other than the study Hospitals. Next to pre-testing, a few modifications were made according to the findings and feedback obtained. The principal investigator supervised the data collection process daily. Since the Epicollect5 application encompasses the JUMP and REQUIRED functions at the design of the questionnaire, data incompleteness and unlikely response issues were not a problem.

### Data processing and analysis

Once the in-patient and post-discharge data collection were completed using Epicollect5 software, the data were downloaded as an Excel file format and then exported to Stata ™ and SPSS software file formats. Descriptive analysis of categorical variables was performed through frequency tables, and mean with SD/median with interquartile ranges were computed for continuous variables. The survival time of SSI was estimated using the Kaplan–Meier (KM) method. The log-rank test was used to compare the estimated survival curve of patients based on categorical variables. The incidence density (rate) and cumulative incidence density was calculated using STATA.

Before running the Cox Proportional hazard regression model, multicollinearity was checked using the variance inflation factor (VIF) for continuous variables. All variables were found to be < 10 with a mean VIF value of 1.05. The proportional hazard assumption (PHA) was checked to identify the combined effects of several covariates on the hazard ratio, using scaled Schoenfeld residual tests (phtest). All variables fulfilled this PHA assumption (*p-value* > 0.05) with the global test value of X^2^ = 31.97, *p-value* = 0.7435. To identify potential predictors of time to development of SSI, a bi-variable Cox proportional regression model was initially fitted for each explanatory variable, and variables with a *P-value* less than 0.05 were included in the multiple Cox regression model. In the multiple Cox analysis, variables that have a *P*-value less than 0.05 were significant predictors of SSI. Since most patients in this data set share the event times, the time was tied and the exact partial likelihood method was used to handle tied events.

### Ethical approval and consent.

Ethical clearance was obtained from the Bahir Dar University, College of Medicine and Health Sciences, Ethical review board (Reference number: *CMHS/IRB 01–008*). A formal letter was submitted and written permission was obtained from the study hospitals. Written informed consent to participate was taken from each study participant before data collection. For children less than 18 years, written consent was obtained from their guardians. Before enrolling participants, the principal investigators had made sure that the patients understood what the research process will involve and acknowledged that this is acceptable. Participants were assured of anonymity regarding the collection of data. Patients who developed SSIs during post-discharge follow-up were linked to either nearby health facilities or their respective study hospitals. Surgeons and Nurses were also assured of anonymity in overall reporting. All experiments and relevant details were conducted under relevant and approved guidelines and approved by the study Hospitals as well as the Bahir Dar University, College of Medicine and Health Sciences, Ethical review board.

## Result

**Fig. 2:**
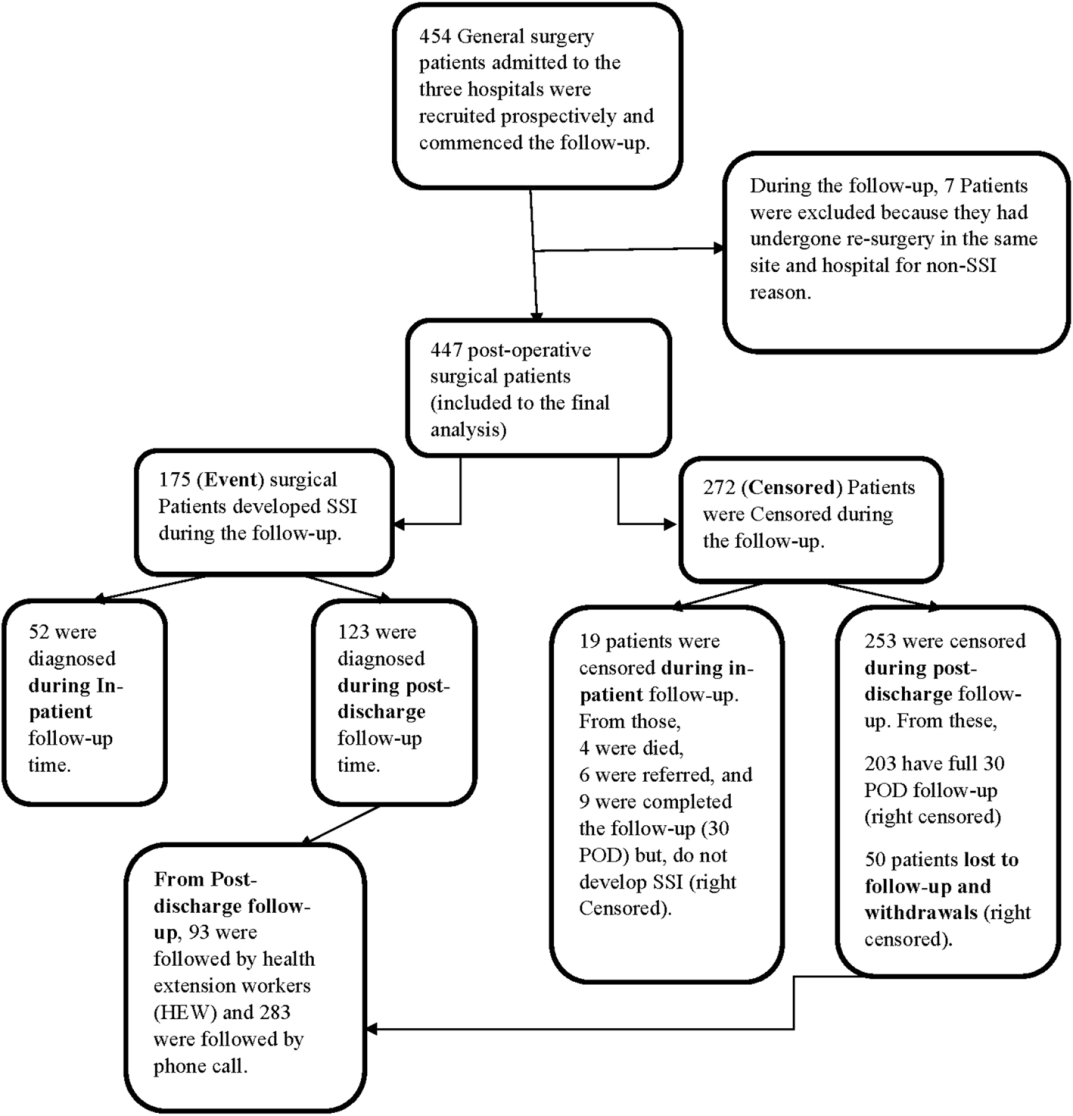
Overall recruitment and follow-up process of surgical patients in Amhara region, Ethiopia, March–April, 2021

At the end of the 30 days of follow-up, 397 (88.8%) participants were known to be either event or censored. From these, 222 (49.6%) of patients were right-censored (did not develop SSI until 30^th^ POD). From the 50 (11.2%) patients who were censored before the 30^th^ POD, 17 (3.8%) did not pick up their phone (lost to follow-up), and 33 (7.4%) their phone did not work after 3 consecutive days of trial. The commonest type of SSI was superficial SSI 128 (73.14%), Deep soft tissue SSI 42 (24%), and organ/Space SSI 5 (2.86%). More than two-thirds of SSI cases (70.3%) occurred at home after patients were discharged from their respective hospitals, while 52 (29.7%) developed in the study hospitals (Fig. 2).

### Socio-demographic characteristics

A total of 447 patients were included in the final analysis, making the response rate 98.6%. The majority of patients were between the age group 41 to 60 years with a mean age of 43.7 ± 13.7 years. Two hundred thirty (51.5%) patients were male and about 226 (50.6%) were rural dwellers. A comparable number of patients were government employees 65 (14.5%) and Housewives 66 (14.8%). Regarding Educational status, 47 (10.5%) of participants were college and above achievers while about 121 (27.1%) were unable to read and write (Table 2).

**Table 2:** Socio-Demographic Characteristics of patients undergone general surgery at specialized hospitals in the Amhara region, between March—April 2021 (*n* = 447)

Variables	Category	Total	
		**Frequency**	**Percent**
Age	< = 20	12	2.7
	21–40	156	34.9
	41–60	232	51.9
	> 60	47	10.5
Sex	Female	217	48.5
	Male	230	51.5
Occupational Status	Private employee	75	16.8
	Government employee	65	14.5
	Merchant	32	7.2
	Farmer	67	15.0
	Pastoralist	31	6.9
	Student	30	6.7
	House Wife	66	14.8
	Unemployed	56	12.5
	Retired	25	5.6
Educational Status	College and Above	47	10.5
	Secondary school	108	24.2
	Primary school	127	28.4
	Able to read and write only	44	9.8
	Unable to read and write	121	27.1
Residence	Urban Resident	221	49.4
	Rural Resident	226	50.6

### Behavioral factors

Regarding behavioral-related factors, the majority of patients 384 (85.9%) do not drink alcohol. Nearly nine-tenths of participants 400 (89.5%) were never smokers while only about 18 (4%) were Current Smokers and the rest were former smokers.

### Patient and surgical wound-related factors

In this study, the mean body mass index (BMI) was 22.35 ± 2.7 kg/m^2^, with 67 (15%) of patients having a BMI > 24.9 kg/m^2^. Seventy-one patients (15.9%) reported having a history of previous surgery and only 38 (8.5%) patients needed a pre-operative blood transfusion. Surgeries classified as Clean-contaminated wound class were 352 (78.7%). The larger number of participants belonged to ASA score I, 179 (40%), and ASA score II, 118 (26.4%) (Table 3).

**Table 3:** Patient and surgical wound-related factors of patients undergone general surgery at specialized hospitals in the Amhara region, between march—April 2021 (*n* = 447)

Variable	Category	Frequency	Percent
History of Previous Surgery	No	376	84.1
	Yes	71	15.9
Preoperative Blood Transfusion	No	409	91.5
	Yes	38	8.5
Surgical Wound Class	Clean	95	21.3
	Clean-contaminated	352	78.7
ASA Score	ASA I	179	40.0
	ASA II	118	26.4
	ASA III	100	22.4
	ASA IV	44	9.8
	ASA V	6	1.4
Body Mass Index	18.5—24.9	355	79.4
	> 24.9	67	15.0
	< 18.5	25	5.6
Length of Pre-Operative Hospital Stay (hours)		**Median**	**IQR**
		32	(18, 73)

### Comorbidity

Patients who had Diabetes Mellitus were 56 (12.5%) while 44 (9.8%) patients had HIV/AIDS. More than two-sixth (153 (35.1%) of the patients had one or more comorbidities such as Hypertension 56 (12.5%), Cancer 46 (10.3%), and/or Asthma 14 (3.1%).

### Hospital and surgical team related factors

The number of surgeries performed by consultant surgeons was 74 (16.6%). Antibiotic Prophylaxis was given to 434 (97.1%) surgical patients and of them, 362 (83.4%) were administered in less than 30 min before the surgery. The median number of professionals attending a particular surgery was 6 (IQR: 6, 8) (Table 4).

**Table 4:** Hospital and surgical team-related factors of patients undergone general surgery at specialized hospitals in the Amhara region, between March—April 2021 (*n* = 447)

Variable	Category	Frequency	Percent
Hospital	FHCSH	145	32.4
	UoGCSH	148	33.1
	TGSTH	154	34.5
The Grade of the Surgeon Who Performed the Surgery	Consultant surgeon	74	16.6
	Surgeon	337	75.4
	Resident	36	8.1
Surgical Antibiotic Given?	Yes given	434	97.1
	No prophylaxis required	11	2.5
	Prophylaxis required but not given	2	0.4
If yes, the time antibiotic was Given (*n* = 434)	Less than 30 min before the surgery	362	83.4
	30 min to 1 h before the surgery	72	16.6
Number of Professionals in the OR while the Surgery was Performed		**Median**	**IQR**
		6	(6, 8)

### Procedure-related Factors

From a total of 447 surgical procedures, a drain was inserted only in 25 (5.6%), and wound care was performed for 406 (90.8%) patients. About two-fifth of procedures (181 (40.5%)) were emergency cases. The median time for the duration of surgery was 1.54 (IQR: 1.24, 2.47) hours.

### Type of surgical procedures

**Fig. 3:**
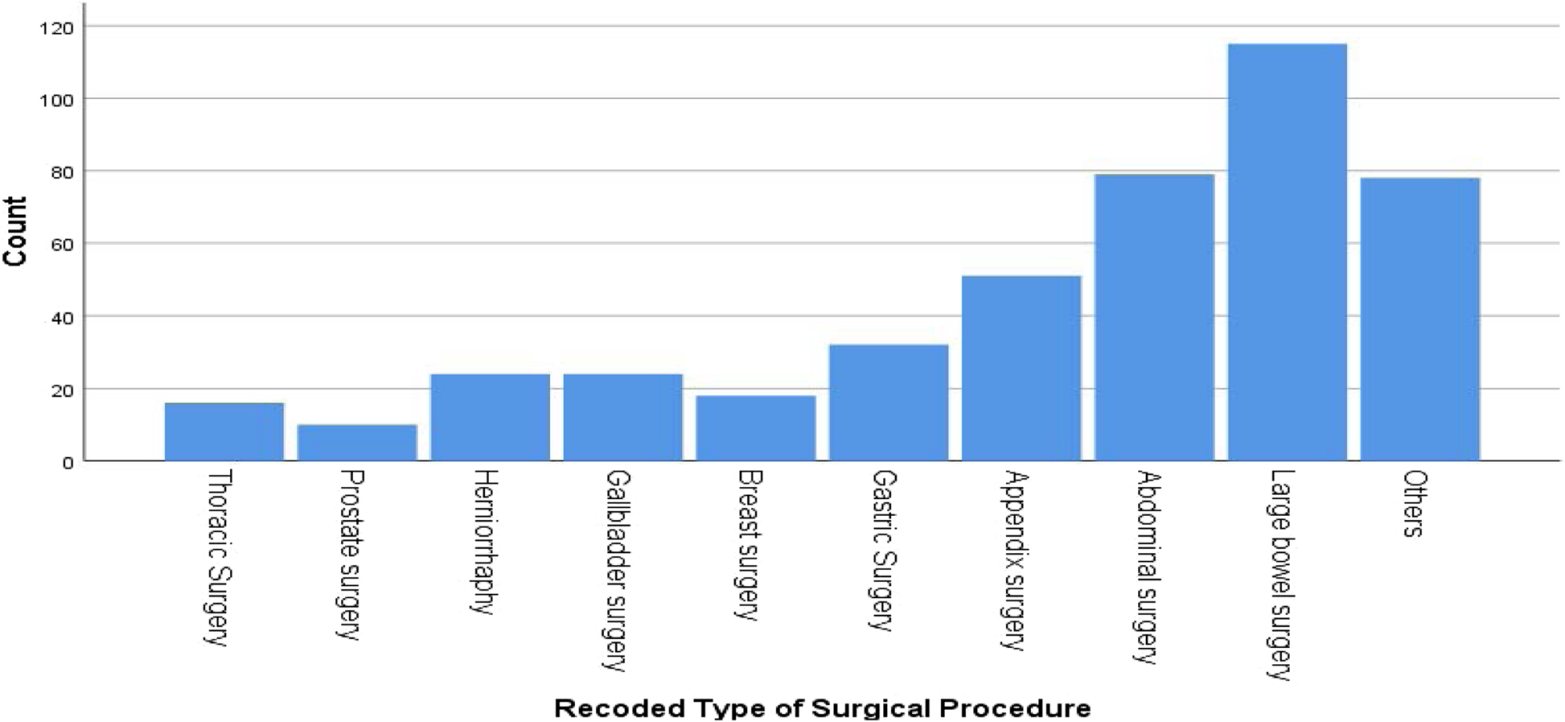
Types of surgical procedures among patients undergone general surgery at specialized hospitals in the Amhara region, between March—April 2021 (*n* = 447)

From all surgical procedures, large bowel surgery was the leading procedure 115 (25.7%) followed by other abdominal Procedures 79 (17.7%), Appendix surgery 51 (11.4%), Gastric Surgery 32 (7.2%), Gallbladder surgery 24 (5.4%), Herniorrhaphy 24 (5.4%), Breast surgery 18 (4%), and others (Fig. 3).

### Incidence of surgical site infection

Patients have been followed up for a total of 9,948 person-days. Among the surgical patients followed, 272 (60.9% [95% CI: 56.2, 65.2]) were censored, while 175 (39.1% [95% CI: 34.7, 43.7]) developed surgical site infections. This makes the cumulative incidence rate 391 per 1000 surgeries. The SSI incidence density in this study was 17.59 (95% CI: 15.1, 20.4) per 1000 person-days of observation.

### Survival time of SSI from date of surgery to 30^th^ POD

**Fig. 4:**
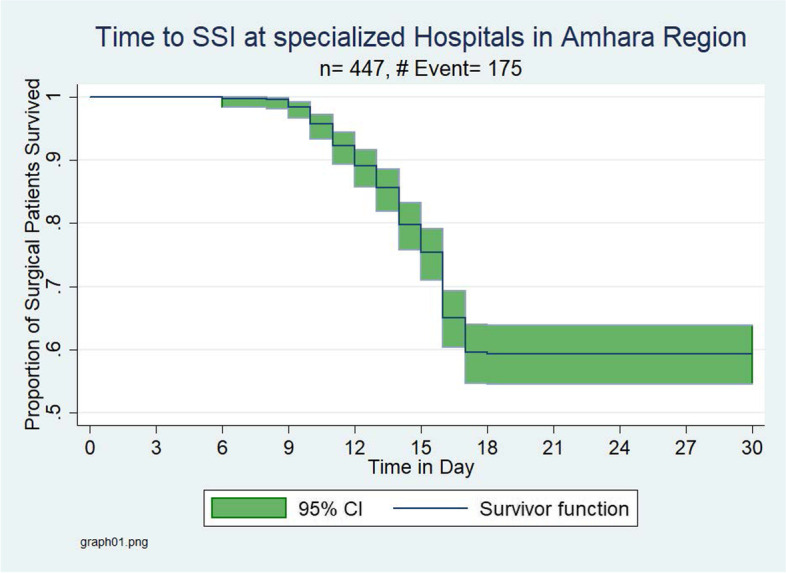
Overall Kaplan–Meier survival estimate for post-operative surgical patients

**Fig. 5:**
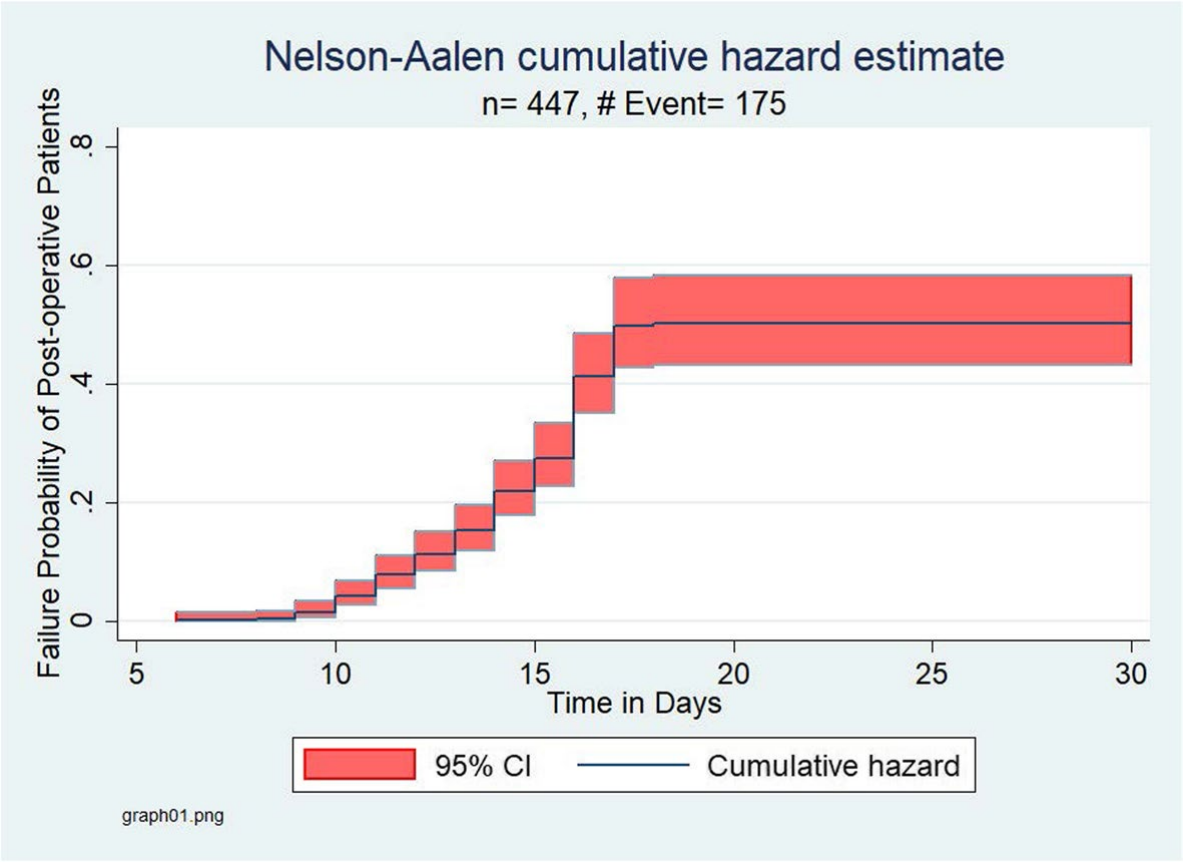
Nelson-Aalen cumulative hazard estimate for post-operative surgical patients

The overall Kaplan–Meier estimate showed that the probability of survival of general surgery patients is high in the first six postoperative days, which falls dramatically as follow-up time increases. But, after 17 post-operative days of follow-up, there was no significant change observed. During the first five days, a maximum (100%) probability of survival was observed and at the end of day six, a 99.7% probability of survival with a standard error of 0.0022 (95%CI: 0.9842–0.9997) was observed. At the end of the 14^th^ day of stay, the probability of survival was found to be 79.79% with a standard error of 0.0193 (95%CI: 0.7570–0.8327), and during the PODs between 18 and 30, the graph becomes straight which indicates the proportion of postoperative surgical patients who survived remained constant, indicating virtually no SSI cases. At the 30^th^ PODs of stay, the cumulative probability of survival of surgical patients was 59.1% with a standard error of 0.0238% (95%CI: 0.5431–0.6364) (Figs. 4–5).

**Fig. 6:**
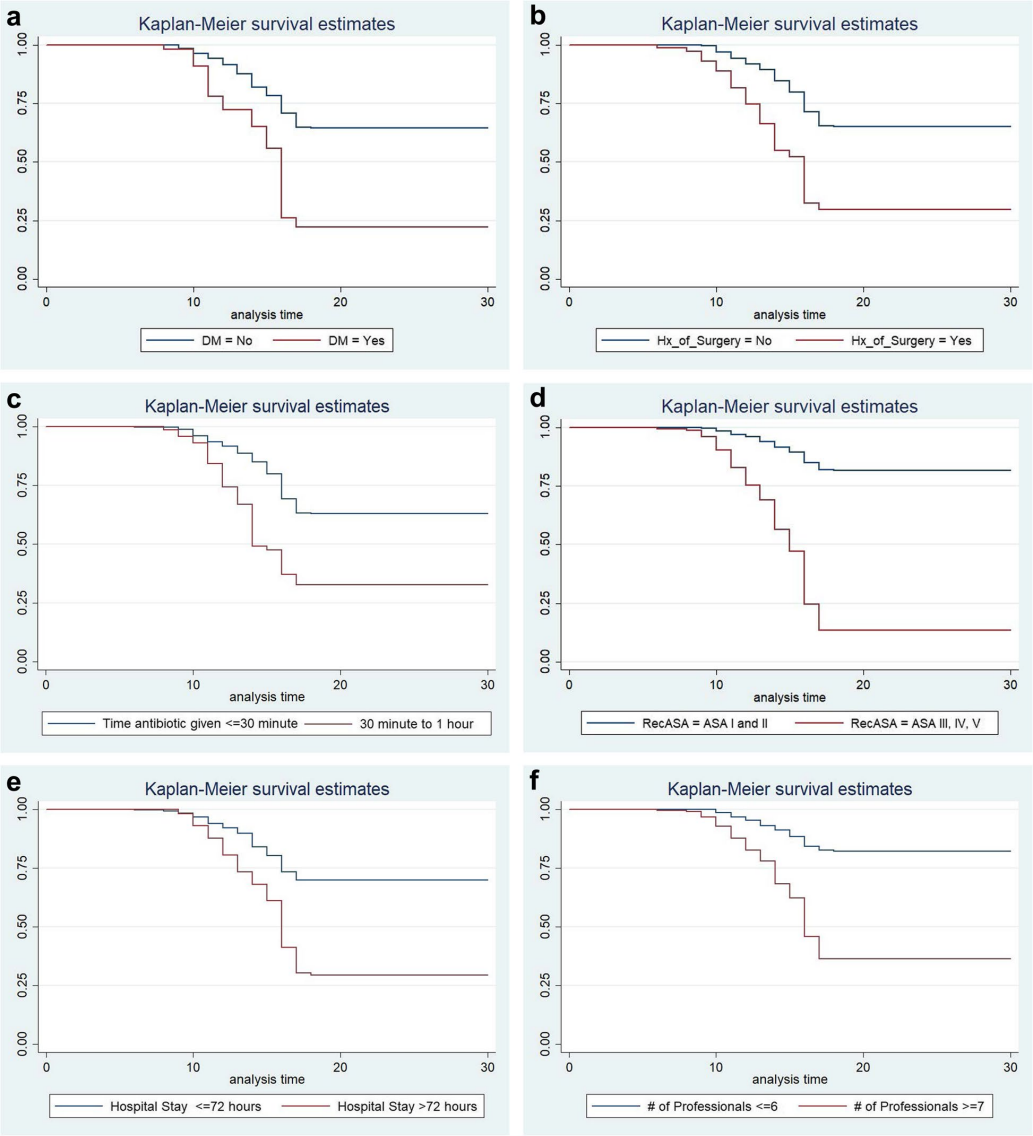
**a** Kaplan–Meier survival curve, comparison of survival time with indifferent categories of Diabetes Mellitus. **b** Kaplan–Meier survival curve, comparison of survival time with indifferent categories of surgical history. **c** Kaplan–Meier survival curve, comparison of survival time with indifferent categories of the timing of antibiotic prophylaxis. **d** Kaplan–Meier survival curve, comparison of survival time with indifferent categories of ASA class. **e** Kaplan–Meier survival curve, comparison of survival time with indifferent categories of length of preoperative hospital stay. **f** Kaplan–Meier survival curve, comparison of survival time within different categories of the number of professionals in the OR

**Fig. 7:**
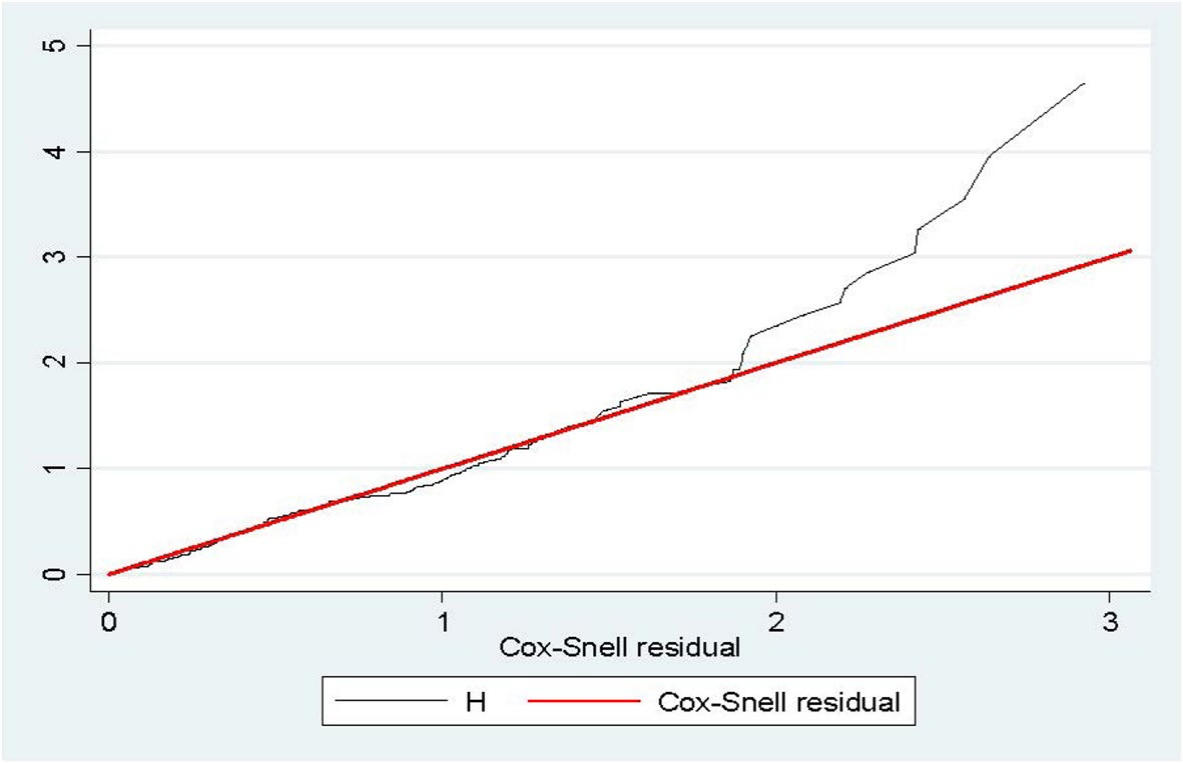
Cox-Snell residual Nelson -Alen cumulative hazard graph on surgical patients at specialized hospitals in Amhara region, Northern Ethiopia, March—April 2021

To test the equality of survival curves of different categorical predictor variables, Cochran– Mantel–Haenszel log-rank test was performed. The test statistics which are obtained from the test showed that there is a significant difference in the survival function (curve) of DM (X^2^ for log-rank test = 43.79, *p* = 0.000), History of Previous Surgery (X^2^ = 45.33, *p* = 0.000), Time antibiotic given (X^2^ = 34.22, *p* = 0.000), ASA-Score (X^2^ = 219.38, *p* = 0.000), Length of pre-operative hospital stay (X^2^ = 57.72, *p* = 0.000), and Number of Professionals in the OR (X^2^ = 91.4, *p* = 0.000) (Fig. 6a - f).Cox-Snell residual and Nelson—Alen cumulative hazard graph indicated the overall model fitness of the data in the Cox Proportional hazards regression model. The hazard function follows the 45° line very closely and it starts to depart when the number of censored patients increases, indicating that residuals have a standard censored exponential distribution with the hazard ratio. Hence, the model is a good fit for the data (Fig. 7).

### Predictors of time to development of surgical site infection

The results of the simple Cox regression analysis revealed that 19 factors were associated with the occurrence of SSI at a *p*-value of ≤ 0.05. After controlling confounders in multiple Cox regression analyses; Age, Sex, DM, history of previous surgery, Timing of antibiotic prophylaxis, ASA score, Length of preoperative hospital stay, Duration of the surgery, and Number of professionals in the OR were found to be independent predictors of surgical site infection at any time t.

The multiple Cox regression analysis revealed that holding other variables constant, relative to female surgical patients, male patients have 1.98 times increased hazard of developing SSI (AHR: 1.98, 95% CI: 1.201 – 3.277) at all times of follow-up. Holding other variables constant, surgical patients who had diabetes mellitus have an 81.9% higher hazard of developing SSI than those who did not have DM (AHR: 1.81, 95% CI: 1.097- 3.016). Patients who received Anti-microbial prophylaxis (AMP) within 30 min to 1 h of skin incision have a 2.6 times greater hazard of developing SSI when compared with patients who had received Anti-microbial prophylaxis (AMP) within 30 min before the surgery (AHR: 2.6, 95% CI: 1.676, 4.039) (Table 5).

**Table 5:** Multiple Cox-proportional hazard model of patients who underwent general surgery at Specialized hospitals in the Amhara region, north-west Ethiopia, march–April, 2021 (*n* = 447)

Predictor	Category	Status		CHR (95% CI)	AHR (95% CI)
		**SSI (%)**	**Censored (%)**		
Sex	Female	61 (34.9)	156 (57.4)	1	1
	Male	114 (65.1)	116 (42.6)	2.021 (1.465, 2.789)	1.98 (1.201, 3.277) *
Occupational Status	Private employee	21 (12)	54 (19.9)	1	1
	Government employee	20 (11.4)	45 (16.5)	1.272 (0.678, 2.387)	1.390 (0.604, 3.195)
	Merchant	10 (5.7)	22 (8.1)	1.313 (0.605, 2.850)	2.331 (0.896, 6.062)
	Farmer	36 (20.6)	31 (11.4)	2.765 (1.580, 4.838)	0.708 (0.314, 1.594)
	Pastoralist	10 (5.7)	21 (7.7)	1.228 (0.5662.664)	0.665 (0.262, 1.684)
	Student	3 (1.7)	27 (9.9)	0.358 (0.105, 1.217)	0.695 (0.175, 2.761)
	House Wife	29 (16.6)	37 (13.6)	1.975 (1.105, 3.528)	1.873 (0.845, 4.150)
	Unemployed	29 (16.6)	27 (9.9)	2.359 (1.317, 4.224)	0.706 (0.330, 1.507)
	Retired	17 (9.7)	8 (2.9))	3.832 (1.950, 7.530)	0.842 (0.347, 2.044)
Educational Status	College and Above	14 (8)	33 (12.1)	1	1
	Secondary school	33 (18.9)	75 (27.6)	0.975 (0.513, 1.856)	0.730 (0.323, 1.651)
	Primary school	45 (25.7)	82 (30.1)	1.083 (0.584, 2.009)	0.756 (0.317, 1.799)
	Able to read and write only	14 (8)	30 (11)	1.060 (0.4942.275)	1.125 (0.400, 3.161)
	Unable to read and write	69 (39.4)	52 (19.1)	2.348 (1.295, 4.257)	0.816 (0.299, 2.226)
Residence	Urban Resident	69 (39.4)	152 (55.9)	1	1
	Rural Resident	106 (60.6)	120 (44.1)	1.772 (1.294, 2.427)	1.484 (0.922, 2.388)
Diabetes Mellitus	Yes	42 (24)	14 (5.1)	3.346 (2.295, 4.878)	1.819 (1.097, 3.016) *
HIVAIDS	Yes	33 (18.9)	11 (4)	3.211 (2.129, 4.843)	1.352 (0.799, 2.28)
Other Comorbidity	Yes	88 (50.3)	65 (23.9)	2.349 (1.723, 3.204)	0.708 (0.466, 1.077)
Alcohol Drinking	Yes	39 (22.3)	24 (8.8)	2.344 (1.604, 3.425)	1.148 (0.688, 1.918)
History of Previous Surgery	Yes	50 (28.6)	21 (7.7)	3.185 (2.236, 4.537	2.078 (1.345, 3.211) *
Grade of the Surgeon	Consultant surgeon	41 (23.4)	33 (12.1)	1	1
	Surgeon	125 (71.4)	212 (77.9)	0.568 (0.392, 0.825)	1.280 (0.773, 2.118)
	Resident	9 (5.1)	27 (9.9)	0.332 (0.158, 0.698)	1.422 (0.540, 3.745)
Surgical wound Class	Clean	28 (16)	67 (24.5)	1	1
	Clean-contaminated	147 (84)	205 (75.4)	1.612 (1.063, 2.445)	0.615 (0.333, 1.136)
Timing of antibiotic prophylaxis	Less than 30 min	129 (73.7)	233 (90)	1	1
	30 min to 1 h	46 (26.3)	26 (10)	2.833 (1.971, 4.072)	2.602 (1.676, 4.039) *
Wound care	Yes	170 (97.1)	236 (86.8)	1	
	No	5 (2.9)	36 (13.2)	0.239 (0.097, 0.589)	0.682 (0.218, 2.136)
ASA	ASA I and II	53 (30.3)	244 (89.7)	1	
	ASA III, IV, V	122 (69.7)	28 (10.3)	10.050 (7.041, 14.346)	6.710 (4.108, 10.960) *
Type of Surgical Procedure	Thoracic Surgery	8 (4.6)	8 (2.9)	1	1
	Prostate surgery	8 (4.6)	2 (0.7)	2.710 (0.945, 7.773)	0.490 (0.130, 1.852)
	Herniorrhaphy	7 (4)	17 (6.3)	0.565 (0.197, 1.616)	0.712 (0.211, 2.406)
	Gallbladder surgery	3 (1.7)	21 (7.7)	0.206 (0.053, 0.800)	0.146 (0.024, 0.869)
	Breast surgery	8 (4.6)	10 (3.7)	0.980 (0.351, 2.732)	0.629 (0.170, 2.319)
	Gastric Surgery	13 (7.4)	19 (7)	0.9732 (0.387, 2.445)	0.717 (0.245, 2.100)
	Appendix surgery	18 (10.3)	33 (12.1)	0.742 (0.311, 1.770)	1.131 (0.378, 3.385)
	Abdominal surgery	31 (17.7)	48 (17.6)	0.814 (0.361, 1.835)	0.872 (0.331, 2.299)
	Large bowel surgery	60 (34.3)	53 (20.2)	1.316 (0.607, 2.853)	0.818 (0.312, 2.147)
	Others	19 (10.9)	59 (21.7)	0.462 (0.195, 1.091)	0.817 (0.304, 2.196)
		**Median**	**IQR**		
Age		43	(35, 53)	1.058 (1.044, 1.072)	1.022 (1.000, 1.043) *
Length of Pre-operative Hospital stay		32	(18, 73)	1.0143 (1.010, 1.018)	1.007 (1.002, 1.013) *
Duration of the Surgery		1.54	(1.24, 2.47)	1.057 (1.030, 1.085)	1.035 (1.001, 1.070) *
Number of Professionals in the OR		6	(6, 8)	1.044 (1.021, 1.068)	1.085 (1.037, 1.134) *

*P-value < 0.05

Moreover, relative to patients who have ASA scores I & II, patients who have ASA scores III, IV, and V have 6.7 times increased hazard of developing SSI (AHR: 6.710, 95% CI: 4.108, 10.9). A one-day increase in pre-operative hospital stay was associated with a 16.8% higher hazard of developing SSI at all times of follow-up (AHR: 1.007 95% CI: 1.002, 1.013). Furthermore, as the age of patients increases by one year and all other variables are held constant, the rate of SSI increases by 2.2% (AHR: 1.022 95% CI: 1, 1.043). Finally, when the number of health professionals, who were in the OR during the surgery, increases by 1 person, the hazard of SSI increases by 8.5% (AHR: 1.085 95% CI: 1.037, 1.134) (Table 5).

## Discussion

This study aimed to assess the survival time and predictors of surgical site infection among general surgery patients in specialized hospitals of the Amhara region. As hypothesized, this study found a relatively highest cumulative incidence rate of surgical site infection (39.1%) than studies in different regions of Ethiopia, such as 19.1% in Hawassa University Referral Hospital [8] and 20.6% in Tikur Anbessa Specialized Hospital [23]. Other African countries like Tanzania [24], and Uganda [25] as well as a few Asian countries [26–28] have also reported lower incidence rates than this study. The possible explanation for the observed increment in the incidence rate of SSI might be interhospital differences in the implementation processes of the recommended infection prevention and control practices during perioperative time as well as the presence of strict infection prevention protocols in some of those countries, such as Uganda and Tanzania [25, 29].

One hundred twenty-three (70.3%) of SSI cases were diagnosed during post-discharge follow-up and it is within the international ranges of 24% in Spain [30], 36.4% in Tanzania [24], and 84% in the USA [31], 87.5% in Canada [32]. This is most likely due to the fact that the median length of hospital stays (LOS) of surgical patients in the three study hospitals ranged from 5 to 6 PODs. And since most infections require at least 7 days to be apparent, it is reasonable to expect most infections to occur after discharge than inpatient [32]. Moreover, the increasing use of ambulatory surgery is related to an extremely shorter length of hospital stay, which in turn leads to most SSIs occurring after discharge than in inpatient.

After adjusting for other variables, this study found that male patients have a 1.98 times higher hazard of developing SSI than female patients. Even though this result differs from some published studies in India [33, 34], it is consistent with those of Pakistan [35], Spain [36], and India [28, 37] where males showed a marked preponderance of developing SSI than females. However, a study conducted in Germany [38] argued that gender differences in surgical site infection rates are not always true when focusing on specific procedures. It reported that male patients undergone abdominal surgery had significantly higher SSI rates compared to female patients, while female patients following hernia, gall bladder, and thyroid surgery had higher SSI-rate than males [38].

The hazard rate of developing SSI for surgical patients with Diabetes Mellitus was 81.9% higher than for patients who do not have DM. This is supported by previous findings in the literature including in Ethiopia [9], the USA [39, 40], India [28, 33, 37], and Pakistan [35]. The strong possibility of perioperative hyperglycemia in DM patients and the respective occurrence of a convenient environment for the growth of bacteria is the most probable justification for the link between DM and SSI. Nevertheless, recent studies [36, 41] reported that Diabetes Mellitus is a strong predictor of SSI even after controlling for hyperglycemia. As a result, it is an independent contributor to SSI risk in another mechanism other than perioperative hyperglycemia [42, 43]. On the other hand, other studies argued that it is not actually diabetes mellitus or hyperglycemia, but the complications of DM (other than hyperglycemia) that increase the risk [36, 44]. Therefore, a wider level study that included DM, hyperglycemia, and diabetic complications is required to pinpoint which predictor is truly contributing to the risk of developing SSI.

In this study, patients who have a history of previous surgery had 2 times a higher risk of developing SSI than those not have surgical history. There are studies in Ethiopia that are in line with this finding [8, 23]. The reason for this association might be the presence of prior exposure to a skin incision which causes a break in the skin barrier and allow resistant microorganisms to enter the body. This is known to create intrinsic differences among patients with and without previous surgical history in their baseline susceptibility to infection. Therefore, apparently immunocompetent individuals (patients without previous surgical history) will be compared to patients who have enhanced susceptibility state at baseline and this is believed to result in the link between the risk of SSI and surgical history [45].

World Health Organization recommends an optimal/ideal timing of antimicrobial prophylaxis for surgical patients must be as close to incision time as possible. Because, the infection risk increases as the time interval between preoperative antibiotics and incision time increases. Our study found that patients who received antibiotic prophylaxis between 30 min to 1 h prior to the surgery have a 2.6 times higher hazard of developing SSI than those who received it within 30 min prior to the surgery. This outcome is consistent with studies done in Ethiopia [23] and Iran [46]. A valid justification for this association could be, when AMP is administered earlier before the skin incision, the tissue concentration of antimicrobial prophylaxis will decrease. As a result, the serum antimicrobial concentration will not be at its therapeutic level to fight against bacterial contamination of the wound during the surgery until the skin is closed [22]. Studies aimed to assess the association between intraoperative antibiotic concentrations and efficacy also further support this justification [47]. Therefore, in surgical procedures where the prophylaxis antibiotics were administered too early before the surgery, health professionals should better consider redosing of antibiotics during the surgery [48].

American Society of Anesthesiologists' score determines the preoperative physical status of patients including their comorbidities. Surgical patients with ASA score ≥ III in this study had a 6.7 times higher hazard of SSI than those with ASA classification ≤ II. This concurs well with studies in India [27, 34], Uganda [25], Rwanda [49], and the USA [40]. On the other hand, a study done in Saudi Arabia [50] and Greece [51] reported that ASA classification was insignificant. This disagreement could be interpreted as a result of inconsistencies when the allocation of the ASA score is done. Because the ASA definitions are only based on the severity of the disease and do not consider patient age, sex, weight, the nature of the surgery, the skill of the anesthetist or surgeon, and the degree of pre-surgical preparation, it is greatly prone to subjectivity. As a result, the magnitude, as well as the direction of the association between SSI and ASA score, might greatly be affected [50]. Therefore, anesthesiologists must take extreme caution when classifying special populations such as trauma, pregnancy, and pediatrics.

Prolonged pre-operative hospital stay was associated with a 16.8% higher hazard of SSI in this study and it is in line with studies conducted in Ethiopia [8, 23], Brazil [10], India [33, 37], and Pakistan [35]. Every space in the hospital is assumed to be contaminated. Patients can get contaminated from cross-contamination between the patient, health professionals, healthcare equipment, and the surrounding environment and air as well [52]. Surprisingly, in contrast to what this study found and what is previously thought, a study in Saudi Arabia [50] revealed a negative association. The differences in the quality of healthcare, hospital ward cleanliness, health experience, and health status, as well as socioeconomic status between countries, might be the reason for this discrepancy [34].

Although the presence of high number of health professional in the operation room has been hypothesized to increase the OR environmental contamination and poses a high risk of SSI, no previous studies in Ethiopia have studied this variable. This study found the presence of greater than 7 professionals in the OR is associated with 2.4 times increased hazard of SSI than surgeries where fewer than 7 professionals are present. This finding is consistent with studies in Ghana [16] and Nigeria [53]. An alternative explanation could be pathogenic microorganisms, from the surgical staff's hands, mucous membranes, and other uncovered areas of the skin that might enter into an open incision of the patient’s body and contaminate the open incision during the surgical procedure [36]. A study done in France reported that a patient’s skin is the direct source of contamination in only 2% of SSI cases and the rest 98% of cases are related to the staff-to-patient cross-transmission and airborne particles contamination during and after the procedure [54]. Considering this, hospitals should provide surgical staff disposable impermeable garments made of non-woven fabric to prevent cross-contamination, should direct them to strictly comply with individual decontamination procedures, and limit the number of professionals in the OR to its minimum required [36].

A one-hour increase in the duration of surgery was associated with a 3.5% increase in the hazard of developing SSI. This is in line with other studies carried out in Ethiopia [8, 23], Nigeria [53], India [34], Pakistan [35], and Iran [46]. The definite mechanism through which prolonged surgical time increases the risk of SSI is not entirely comprehended. Even though, it is forecasted that longer operative time would expose patients’ incisions to the environment for an extended period. As a result, the risk of pathogenic microorganism contamination increases [48]. A further important rationalization might be the longer the surgery duration is, the more likely the surgical team would get fatigued and commit technical errors (such as breaking aseptic techniques) that may put the patient at a higher risk of getting contamination [25]. Hence, hospitals should dedicate efforts and resources to reduce operative time. This might include strategies such as the adoption of new technologies that can help improve operative efficiency, utilization of specialized care teams, and ensuring that operative staff is not overworked or fatigued [48].

## Limitations and strengths of the study

The first notable limitation of this study is using telephone follow-up and interview, instead of a physical visit, as a diagnostic tool for post-discharge SSI. However, a systematic review and meta-analysis [55] reported this method has 97.0% sensitivity and 97.7% specificity for detecting SSI. Hence, it provides a practical alternative to clinic-based diagnosis and physical visits. Additionally, a reasonable, but relatively small sample size was used, due to time limitations (the prospective nature of the study) and financial constraints. Moreover, the study was unable to investigate the significant relationship of variables related to; health professionals, antiseptic solutions used for patient preparation, equipment sterilization, and type of anesthesia used. However, the choice of all the predictors included was based on the recommendations in the field and WHO. The first methodological strength of this study lies in the presence of post-discharge follow-up of surgical patients, which is very critical to determine the timing of the infections and the true rate of the problem. In addition, the multicenter-ness of the study increased the generalizability of the results, and finally, since the data were collected using the epicollect5 data collection tool, the data collection was entirely complete and reliable.

## Conclusion and recommendations

The incidence of SSI was higher than the acceptable international range. The majority of the SSIs occurred after hospital discharge and within 16 postoperative days. The main predictors of time to development of SSI were age, sex, DM, previous surgical history, the timing of AMP, ASA score, preoperative hospital stay, duration of surgery, and the number of professionals in the OR. Our findings strongly emphasize hospitals to consider initiating a systematic, complete, and independent post-discharge follow-up program for post-operative patients. This helps not only to determine the true rate of post-discharge SSI, but also to detect cases of SSI as early as possible, to initiate treatments accordingly, and to prevent pressing and fatal complications. Hospitals should also use the evidence (survival time) from this study to plan their postoperative appointment guidelines and policies.

Health professionals should take measures that could reduce modifiable predictors in there day to day practice. These measures include but are not limited to; preoperative planning to avoid unnecessary longer surgery time; adoption of new technologies that can help improve operative efficiency; improving surgeon’s experience; avoiding unnecessary preoperative hospital admissions, avoiding intra-operative teaching or limiting the teaching solely to low-risk operations; antibiotic re-dosing during the procedure; strict adherence to infection prevention measures; keeping the cleanliness and hygiene of the hospital wards; and pre-planning of necessary surgical equipment in case of patients with unexpected longer surgery times.

Finally, this study recommends further studies in Ethiopia regarding the diagnostic accuracy of using telephone calls for post-discharge detection of SSIs, as compared to the gold standard (direct observation by health professionals). We also recommend further research, which includes all Diabetes Mellitus, its complications, and perioperative hyperglycemia, to be undertaken to determine the specific contributor that increases the hazard of developing SSI.

## Data Availability

The datasets used during the current study are available upon reasonable request to the corresponding author.
